# Short-range C-signaling restricts cheating behavior during *Myxococcus xanthus* development

**DOI:** 10.1128/mbio.02440-24

**Published:** 2024-10-18

**Authors:** Y. Hoang, Joshua Franklin, Yann S. Dufour, Lee Kroos

**Affiliations:** 1 Department of Biochemistry and Molecular Biology, Michigan State University, East Lansing, Michigan, USA; 2 Department of Microbiology and Molecular Genetics, Michigan State University, East Lansing, Michigan, USA; Max-Planck-Institut fur terrestrische Mikrobiologie, Marburg, Germany

**Keywords:** cheating, extracellular signaling, *Myxococcus xanthus*, bacterial development, spores, biofilms, fruiting body, short-range signaling, multicellular development, evolution

## Abstract

*Myxococcus xanthus* uses short-range C-signaling to coordinate multicellular mound formation with sporulation during fruiting body development. A *csgA* mutant deficient in C-signaling can cheat on wild type (WT) in mixtures and form spores disproportionately, but our understanding of cheating behavior is incomplete. We subjected mixtures of WT and *csgA* cells at different ratios to co-development and used confocal microscopy and image analysis to quantify the arrangement and morphology of cells. At a ratio of one WT to four *csgA* cells (1:4), mounds failed to form. At 1:2, only a few mounds and spores formed. At 1:1, mounds formed with a similar number and arrangement of WT and *csgA* rods early in development, but later the number of *csgA* spores near the bottom of these nascent fruiting bodies (NFBs) exceeded that of WT. This cheating after mound formation involved *csgA* forming spores at a greater rate, while WT disappeared at a greater rate, either lysing or exiting NFBs. At 2:1 and 4:1, *csgA* rods were more abundant than expected throughout the biofilm both before and during mound formation, and cheating continued after mound formation. We conclude that C-signaling restricts cheating behavior by requiring sufficient WT cells in mixtures. Excess cheaters may interfere with positive feedback loops that depend on the cellular arrangement to enhance C-signaling during mound building. Since long-range signaling could not likewise communicate the cellular arrangement, we propose that C-signaling was favored evolutionarily and that other short-range signaling mechanisms provided selective advantages in bacterial biofilm and multicellular animal development.

**IMPORTANCE:**

Bacteria communicate using both long- and short-range signals. Signaling affects community composition, structure, and function. Adherent communities called biofilms impact medicine, agriculture, industry, and the environment. To facilitate the manipulation of biofilms for societal benefits, a better understanding of short-range signaling is necessary. We investigated the susceptibility of short-range C-signaling to cheating during *Myxococcus xanthus* biofilm development. A mutant deficient in C-signaling fails to form mounds containing spores (i.e., fruiting bodies) but cheats on C-signaling by wild type in starved cell mixtures and forms spores disproportionately. We found that cheating requires sufficient wild-type cells in the initial mix and can occur both before mound formation and later during the sporulation stage of development. By restricting cheating behavior, short-range C-signaling may have been favored evolutionarily rather than long-range diffusible signaling. Cheating restrictions imposed by short-range signaling may have likewise driven the evolution of multicellularity broadly.

## INTRODUCTION

Microbiomes often contain bacteria that adhere to biotic and abiotic surfaces, forming biofilms that affect ecosystems and human health in diverse and important ways (1–3). Within biofilms, bacteria communicate using both long- and short-range signaling mechanisms (4). Long-range signaling involves the release of diffusible signal molecules from cells and does not require cell–cell contacts (5). Short-range signaling typically depends on cell-surface-associated protein assemblies that mediate direct cell–cell contact (6). Both long- and short-range signaling shape the composition, spatial structure, ecology, and evolution of biofilms (7–9). A better understanding of the mechanisms and functions of signaling interactions within biofilms will facilitate their manipulation for societal benefits (3, 10) and provide insights into the evolution of multicellularity (11, 12).

Signaling often promotes cooperation between individuals but exposes the community to exploitation by cheaters, which reduce or eliminate the production of the signal molecule but gain a fitness advantage by responding to the signal molecule produced by cooperators (13). Cheating is pervasive in microbial communities, and its consequences can be profound (e.g., community collapse), so cooperators evolve to combat cheating (8, 9, 14, 15). Efforts to manipulate battles between cooperators and cheaters for therapeutic, agricultural, industrial, and environmental applications are gaining traction (10, 16–22).

In this study, we used a biofilm formed by *Myxococcus xanthus* as a model to investigate cheating on short-range C-signaling. *M. xanthus* adheres to the bottom of a container and forms a biofilm submerged under a thin layer of liquid (23). In the absence of nutrients, the cells coordinate their movements to build dome-shaped mounds, which mature into fruiting bodies as some of the rod-shaped cells differentiate into round spores. Other rods lyse or remain outside fruiting bodies as peripheral rods (24, 25). During the developmental process, C-signaling coordinates mound formation with spore differentiation (26–28). A *csgA* mutant deficient in C-signaling fails to build mounds or form spores (29–31). Upon co-development with an equal number of wild-type cells, *csgA* mutants have been reported to form an approximately equal number of spores as the WT (29, 32) or ~100-fold (33) to ~380-fold (34) more spores than the WT. An equal number of spores indicates rescue of *csgA* development by WT C-signaling. A greater number of *csgA* than WT spores indicates developmental cheating by *csgA* on WT C-signaling. Clearly, *csgA* mutants respond to WT C-signaling, but our understanding of the requirements for rescue and cheating behavior is incomplete.

Neither are the C-signal production and reception mechanisms completely understood (reviewed in reference 35). In one model, starving cells secrete a protease (36, 37) that cleaves CsgA to a 17-kDa fragment (p17) at the surface of producer cells (38, 39), and responders detect p17 with an unidentified cell-surface receptor. In another model, starving cells synthesize intact 25 kDa CsgA with cardiolipin phospholipase enzymatic activity that releases diacylglycerols from the inner membrane (40), but how these signal molecules exit producer cells and how responders perceive them are unknown. The two models are not mutually exclusive (i.e., CsgA might be bifunctional).

Although gaps remain in our molecular understanding of C-signaling, knowledge continues to grow about the cellular requirements for efficient C-signaling. Early work indicated that C-signaling requires cells to be in close proximity, possibly in contact (38), and that cell motility and alignment increase C-signaling (41–43). Recently, tracking of *csgA* mutant cells mixed with a 10,000-fold excess of WT cells revealed complete rescue of *csgA* participation in mound formation, despite differences from WT in motility behavior (primarily, faster speeds of *csgA* rods compensated for their weaker bias in the persistent duration of movement toward nascent mounds) (44). C-signaling also affects the expression of many genes during development (32), apparently by activating the transcription factor FruA posttranslationally (45, 46), although the mechanism is unknown. We recently used confocal microscopy and cell segmentation to visualize and quantify C-signal-dependent gene expression of cells within 5–10 µm of the bottom of NFBs (47), which mature to a height of ~50 µm. We found that expression in transitioning cells (TCs) (i.e., cells intermediate in morphology between rods and spores) and spores later in development correlated with earlier cell density, alignment of neighboring rods, and tangential orientation of rods, suggesting that the arrangement of cells within NFBs affects the efficiency of C-signaling, which regulates the spatiotemporal patterns of gene expression and cellular differentiation.

To investigate the cheating behavior of a *csgA* mutant deficient in C-signaling, we mixed the mutant with WT cells proficient at C-signaling. We induced mixtures at different ratios to co-develop and used our new methods of visualizing and quantifying the arrangement and morphology of cells near the bottom of the biofilm. Our results show that cheating by *csgA* on WT C-signaling requires sufficient WT cells in the initial mixture and can occur both before and after the mound-building stage of development. By restricting cheating behavior to specific initial cell ratios, short-range C-signaling may have provided a selective advantage evolutionarily. We discuss how cheating restrictions imposed by short-range signaling may have likewise led to their prevalence in biofilm and animal development.

## RESULTS

### Wild type and a *csgA* mutant co-develop well when mixed 1:1 but not when mixed 1:2

To visualize WT and *csgA* mutant cells during co-development, we engineered strains to produce different fluorescent proteins under the control of a vanillate-inducible promoter. In control experiments, we mixed each strain with its unlabeled parent at a ratio of 1:5 to allow visualization of individual labeled cells and added vanillate at the beginning of starvation. As expected (47), the WT mixture formed mounds by 24 h poststarvation (PS) and many fluorescent spores were observed at 42 h in images of optical sections acquired near the bottom of the same NFB using confocal laser scanning microscopy (Fig. S1). In contrast and as expected (29–31), the *csgA* mutant mixture failed to form NFBs (Fig. S1).

**Fig 1:**
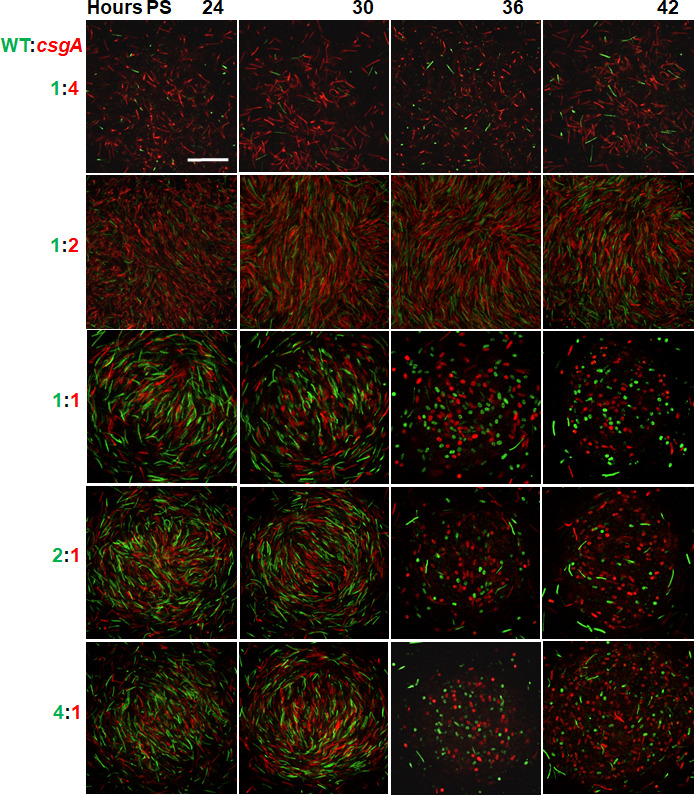
Co-development of wild type (WT) and *csgA* mutant cells at different ratios. WT and *csgA* cells were mixed at ratios indicated on the left. Half of the WT cells were the labeled strain YH7 (green fluorescence), and half were the unlabeled strain DK1622. Likewise, half of the *csgA* cells were the labeled strain YH11 (red fluorescence), and half were the unlabeled strain MRR33. Vanillate (0.5 mM) was added, and the mixtures were starved under submerged culture conditions. Confocal images of the same field of view were acquired near the bottom (i.e., the first optical section above the bottom of the well in which cells could be clearly visualized, so ~0.25 to 0.5 µm above the bottom of the well) of the biofilm or a mound at the indicated times poststarvation (PS). Images show the green and red channels merged and are representative of five biological replicates. Bar, 20 µm.

To allow visualization of individual-labeled cells in co-development experiments, we mixed each labeled strain 1:1 with its unlabeled parent, then mixed the WT and *csgA* mutant cells over a range of ratios, and added vanillate at the beginning of starvation. Mixtures of WT and *csgA* at 1:4 failed to form mounds (Fig. 1; Fig. S2 and S3). At 1:2, a few mounds formed by 24 h PS (Fig. 1; Fig. S3) and persisted until 42 h (Fig. 1), but very few spores formed (Fig. 1; Fig. S2). Strikingly, at ≥1:1 (i.e., equal or excess WT cells), mounds formed by 24 h (Fig. 1; Fig. S3), and TCs and numerous spores formed by 36 h (Fig. 1; Fig. S2). At 2:1 and 4:1, red fluorescent *csgA* spores appeared to be more numerous than expected at 36 and 42 h, suggestive of *csgA* cheating. We also observed a few red fluorescent TCs and spores at 30 h in those mixtures (Fig. S2), but not green fluorescent ones, suggesting that *csgA* was ahead of WT in development. The mixtures at 72 and 96 h looked similar to the 42 h mixtures (data not shown). As a control experiment, we grew strains in the presence of vanillate, made mixtures at ≥1:2 as described above, and immediately counted labeled cells using confocal microscopy. The observed ratios of WT to *csgA* cells were similar to the expected ratios (Fig. S4). We conclude that development requires sufficient WT cells in the initial mix, and our qualitative observations suggest that at ≥2:1, *csgA* spores form disproportionately as NFBs mature.

### Mixtures initially at ≥1:1 form NFBs with normal cell density, alignment, and orientation

**Fig 2:**
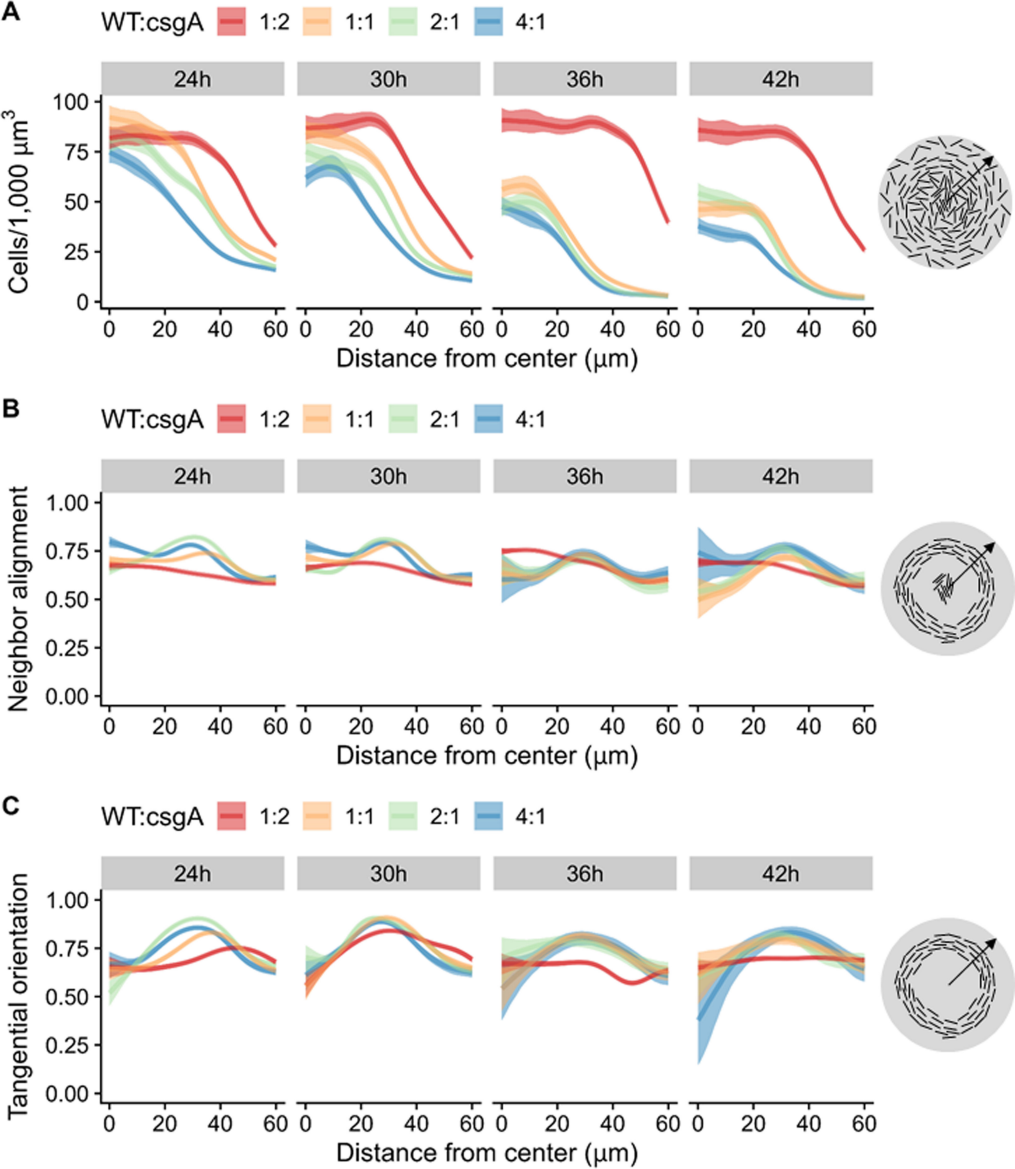
Radial patterns of cell density, neighbor alignment, and tangential orientation in co-developed mixtures at different times poststarvation. In the experiment described in the Fig. 1 legend, *z*-stacks of optical sections were collected from near the bottom of the same nascent fruiting body (NFB) to 5 µm up for each of the five biological replicates, and segmented cells were classified as rods, transitioning cells, or spores. The combined results for both WT green- and *csgA* red-labeled cells are shown. Line, median. Shaded region, 90% credible interval. (**A**) Combined density of all cell classes from the center (0 µm) to the edge (60 µm) of NFBs. The cartoon at the right depicts an early NFB (gray circle) with decreasing rod density ~30–60 µm from the radial center (arrow, radius). (**B**) Neighbor alignment of rods radially in NFBs over time. Alignment is the weighted average of the cosine of the angle of a rod to each of its neighbors (using a Gaussian kernel with sigma = 2.5 µm) (1, perfect alignment; 0, orthogonal). Cartoon, early NFB with maximal neighbor alignment near the radial center and ~20–40 µm from the center. (**C**) Tangential orientation of rods radially in NFBs over time. Orientation is the cosine of the angle of a rod with the circumference of the NFB (1, tangent to the circumference; 0, orthogonal). Cartoon, early NFB with maximum tangential orientation ~20–40 µm from the center.

Radial patterns of cell density, alignment of neighboring rods, and tangential orientation of rods in NFBs early during WT development correlate with C-signal-dependent gene expression and spore proportion later as NFBs mature (47). For comparison with WT, we quantified the arrangement and morphology of labeled cells in the co-developed mixtures of WT and *csgA* initially at ≥1:2, which formed mounds (Fig. 1; Fig. S3). Computational analysis of *z*-stacks of optical sections collected from near the bottom of NFBs to 5 µm up (i.e., several cell layers since rods, TCs, and spores are ~0.3–0.6 µm in width) (47) led to three-dimensional segmentation of cells in image stacks and classification of individual cells as rods, TCs, or spores. Because we modified the computational analysis to improve cell detection and segmentation in the new confocal images, we also re-analyzed the WT *z*-stacks collected previously (47). Mixtures initially at ≥1:1 formed NFBs with high cell density within 20 µm of the radial center by 30 h PS and lower cell density later (Fig. 2A), consistent with WT alone (Fig. S5A). At 1:2, the mounds exhibited a larger radius of high cell density, which did not decrease by 42 h (Fig. 2A), suggesting that less cell lysis occurred, as observed previously for *csgA* mutants alone (48–50).

For all the mixtures, most of the cells were rods at 24 and 30 h PS (Fig. S6), and the rods were most aligned with each other near the radial center (Fig. 2B), consistent with WT alone (Fig. S5B). Most of the cells remained rods in the mounds formed by the 1:2 mixture, but in the NFBs formed by mixtures initially at ≥1:1, most of the cells became spores located within ~20 µm of the radial center by 36 h (Fig. S6), consistent with WT aIone (Fig. S7A). The mixtures initially at ≥1:1 resembled WT alone in terms of the tangential orientation of rods, which was greatest at ~20 to 40 µm from the radial center of NFBs at 24 and 30 h (Fig. 2C; Fig. S5C).

Altogether, the results indicate that mixtures initially at ≥1:1 form NFBs with normal radial patterns of rod density, alignment, and orientation early, and a normal radial pattern of spores at the normal time.

### Cheating can occur both early and later in development

**Fig 3:**
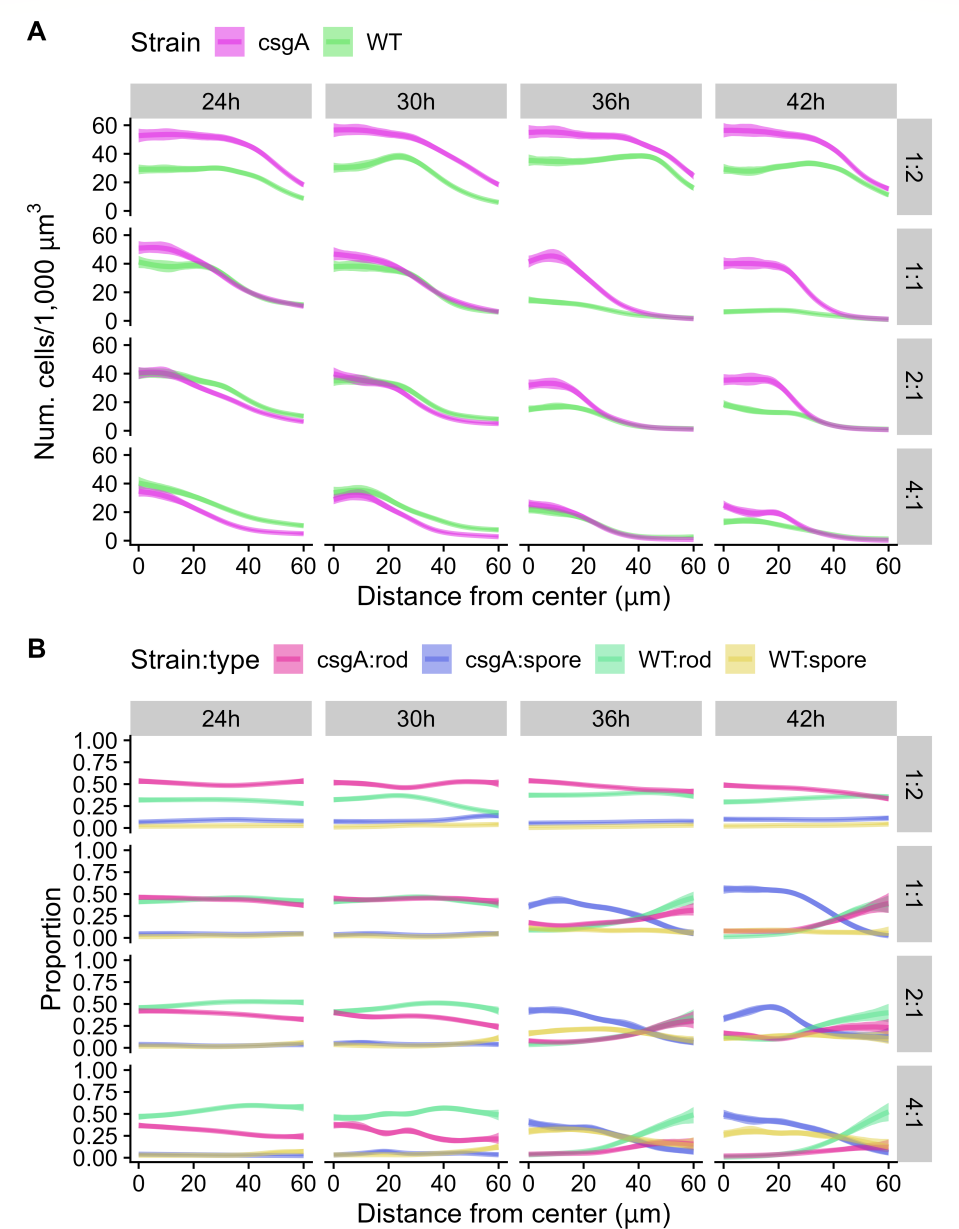
Radial patterns of wild type (WT) and *csgA* mutant cells in co-developed mixtures at different times poststarvation. In the experiment described in the Fig. 1 and 2 legends, segmented cells from *z*-stacks were classified as rods, transitioning cells, or spores. The results for WT green- and *csgA* red-labeled cells are shown separately. (**A**) Combined density of all cell classes from the center (0 µm) to the edge (60 µm) of nascent fruiting bodies (NFBs). (**B**) Proportion of WT and *csgA* mutant rods and spores (relative to the combined total number of cells in all classes for both strains) radially in NFBs over time. Line, median. Shaded region, 90% credible interval.

We compared the radial patterns of WT and *csgA* mutant cells in the co-developed mixtures. Strikingly, *csgA* was more abundant than expected in NFBs formed at 24 and 30 h PS by mixtures initially at ≥2:1 (Fig. 3A). In these early NFBs, most of the cells were rods (Fig. S6). Yet, despite a twofold or even a fourfold excess of WT rods initially (Fig. S4), the *csgA* cell density was similar to that of WT across the radii of these NFBs (Fig. 3A), indicative of *csgA* cheating on WT C-signaling early in development. The *csgA* cell density was also similar to that of WT in early NFBs formed by the 1:1 mixture, indicative of efficient rescue of *csgA* mound formation by WT C-signaling but not cheating by *csgA*. Likewise, the initial twofold excess of *csgA* rods in the 1:2 mixture appeared to persist in early NFBs, suggesting at least partial rescue of mound formation. The radial patterns of WT and *csgA* rods did not differ significantly in terms of neighbor alignment and tangential orientation in the co-developed mixtures (Fig. S8).

In the maturing NFBs at 36 and 42 h PS, most of the cells had become spores for the mixtures initially at ≥1:1 (Fig. S6). For the 1:1 and 2:1 mixtures, the *csgA* cell density exceeded that of WT near the center, supportive of cheating during sporulation, since the *csgA* cell density was similar to that of WT at earlier times (Fig. 3A). For the 4:1 mixture, the *csgA* cell density was similar to that of WT at 36 h and slightly greater than that of WT near the center at 42 h. Our observations indicated that for mixtures initially at ≥1:1, many *csgA* rods became spores, but owing to the presence of some rods and TCs, further analysis was necessary to determine whether cheating occurred during the sporulation stage of development.

To compare the radial patterns of WT and *csgA* mutant rods, TCs, and spores in the co-developed mixtures, we calculated the proportion of each cell class. The proportions at 24 and 30 h PS show that *csgA* rods were more abundant than expected (based on the initial ratio of the two strains) in early NFBs formed by mixtures initially at ≥2:1, especially closer to the center (Fig. 3B). We conclude that cheating occurs before and/or during mound formation in those mixtures. The proportion of TCs was low at all times for all the co-developed mixtures (Fig. S9), as observed for WT alone (47) (Fig. S7B); however, the proportion of TCs was slightly greater for WT alone at 30 h (Fig. S7B) than the mixtures (Fig. S9), suggesting that *csgA* delays WT development slightly in the mixtures. In the maturing NFBs formed by mixtures initially at ≥1:1, the proportions of *csgA* TCs and spores exceeded or equaled those of WT at 36 and 42 h (Fig. 3B; Fig. S9). We conclude that cheating occurs during sporulation in mixtures initially at ≥1:1.

**Fig 4:**
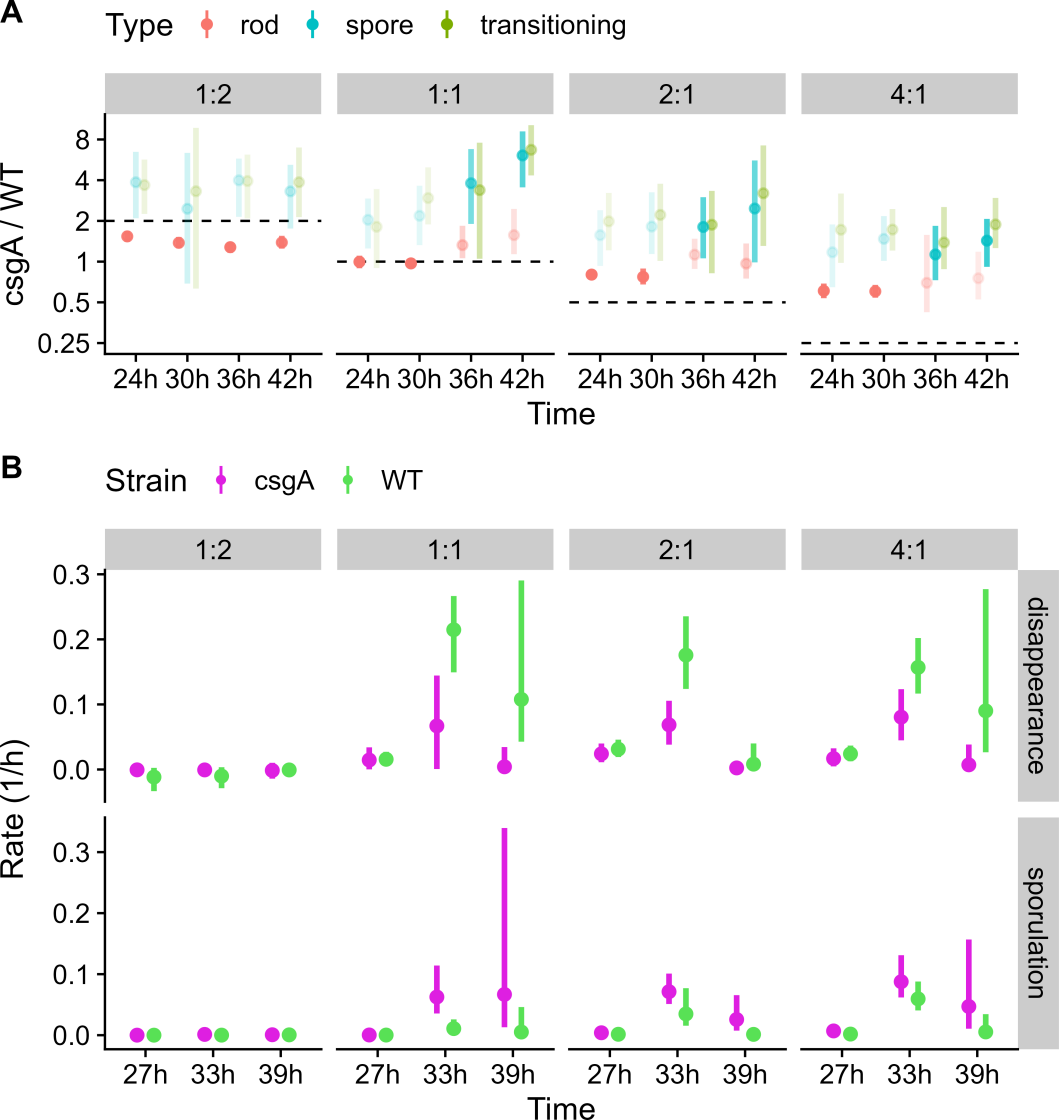
Ratios of *csgA* to wild type (*csgA*/WT) cell classes and rates of disappearance and sporulation in co-developed mixtures over time. In the experiment described in Fig. 1 and 2 legends, segmented cells from *z*-stacks were classified as rods, spores, or transitioning cells (transitioning). Cells within 60 µm of the radial center of nascent fruiting bodies were analyzed. (**A**) Ratios of *csgA*/WT cell classes at indicated times poststarvation (PS). Bold color, major cell class. Pale color, minor cell class. Dashed line, initial ratio. (**B**) Rates of *csgA* and WT disappearance and sporulation during 6-h intervals PS. Time, interval halfway point. Dot, median. Line, 90% credible interval.

To quantify cheating in the co-developed mixtures irrespective of the radial patterns, we compared the observed ratios of *csgA* to WT (*csgA*/WT) rods, TCs, and spores with the initial ratios. In Fig. 4A, the major cell class observed at each time point is shown in bold color and the minor cell classes in pale colors, and dashed lines show the initial ratios. In agreement with our conclusion from the radial patterns, the *csgA*/WT ratio of rods exceeded the initial ratio in NFBs formed by the 2:1 and 4:1 mixtures at 24 and 30 h PS (*P* < 10^−4^), showing the extent of cheating before and/or during mound formation. The observed ratios of TCs and spores further exceeded the initial ratio at 36 and 42 h for those mixtures (*P* < 10^−4^), showing that cheating continued during the sporulation stage of development. For the 1:1 mixture, cheating occurred only during the sporulation stage of development (*P* < 10^−4^), also in agreement with our conclusion from the radial patterns. Interestingly, the *csgA*/WT ratio of TCs and spores was about fourfold greater than the initial ratio for all three mixtures in which cheating occurred. For the 1:2 mixture, the observed ratios of rods were less than the initial ratio (*P* < 10^−4^), showing that C-signaling by WT failed to fully rescue participation of *csgA* in mound formation.

### Cheating after mound formation involves the *csgA* mutant forming spores at a greater rate than WT

To quantify cheating in the co-developed mixtures during the spore-forming stage of development after 24 h PS, we calculated the rates of *csgA* and WT “disappearance” and sporulation during 6-h intervals between time points. To limit the number of unknown parameters in the rate equations, we grouped the numbers of rods and TCs together since either could “disappear” due to lysis, motile rods could “disappear” by exiting NFBs, and TCs could “disappear” by completing the transition to spores. As a result, the change in spore and rod/TC numbers were monotonic over time (Fig. S10), allowing us to estimate the rates of change for each time interval. For mixtures initially at ≥1:1, the rate of *csgA* sporulation exceeded that of WT on average during the 30- to 36-h and 36- to 42-h intervals designated 33 and 39 h, respectively (Fig. 4B) (*P* = 0.039). Conversely, the rate of WT “disappearance” exceeded that of *csgA* on average during the 33- and 39-h intervals for mixtures initially at ≥1:1 (*P* = 0.013). Since the greater “disappearance” of WT is not due to TCs becoming spores, it must be due to lysis of TCs or rods, or rods exiting NFBs. We conclude that cheating after mound formation involves *csgA* forming spores at a greater rate than WT, which “disappears” at a greater rate by lysing or exiting NFBs.

### Cheating can occur before mound formation due to greater survival of the *csgA* mutant

The cheating we observed in early NFBs formed by mixtures initially at ≥2:1 could involve differential survival and/or movement of *csgA* and WT. Greater survival of *csgA* could result in cheating throughout the biofilm, whereas greater movement of *csgA* into mounds and/or WT out of mounds could result in the opposite of cheating outside of mounds (i.e., *csgA*/WT < initial ratio). To compare the composition of the early NFBs to their surroundings, we acquired *z*-stacks of confocal images from near the bottom to 5 µm up at 24 and 30 h PS. We also imaged the biofilm at 18 h prior to the formation of most mounds (Fig. S11). We mixed each labeled strain with its unlabeled parent at 1:3 for the 18- and 24h time points or 1:1 for the 30-h time point, then mixed the WT and *csgA* cells at different ratios for co-development in the presence of vanillate. The 1:3 proportion of labeled cells in the mixtures did not alter co-development at different ratios at 24 h (see below), but was necessary at 18 h to facilitate cell segmentation and classification because of the higher cell density, based on preliminary experiments. The 1:1 proportion of labeled cells in the mixtures was used for the 30-h time point in order to visualize more labeled cells outside of NFBs, where cell numbers decline (see below).

**Fig 5:**
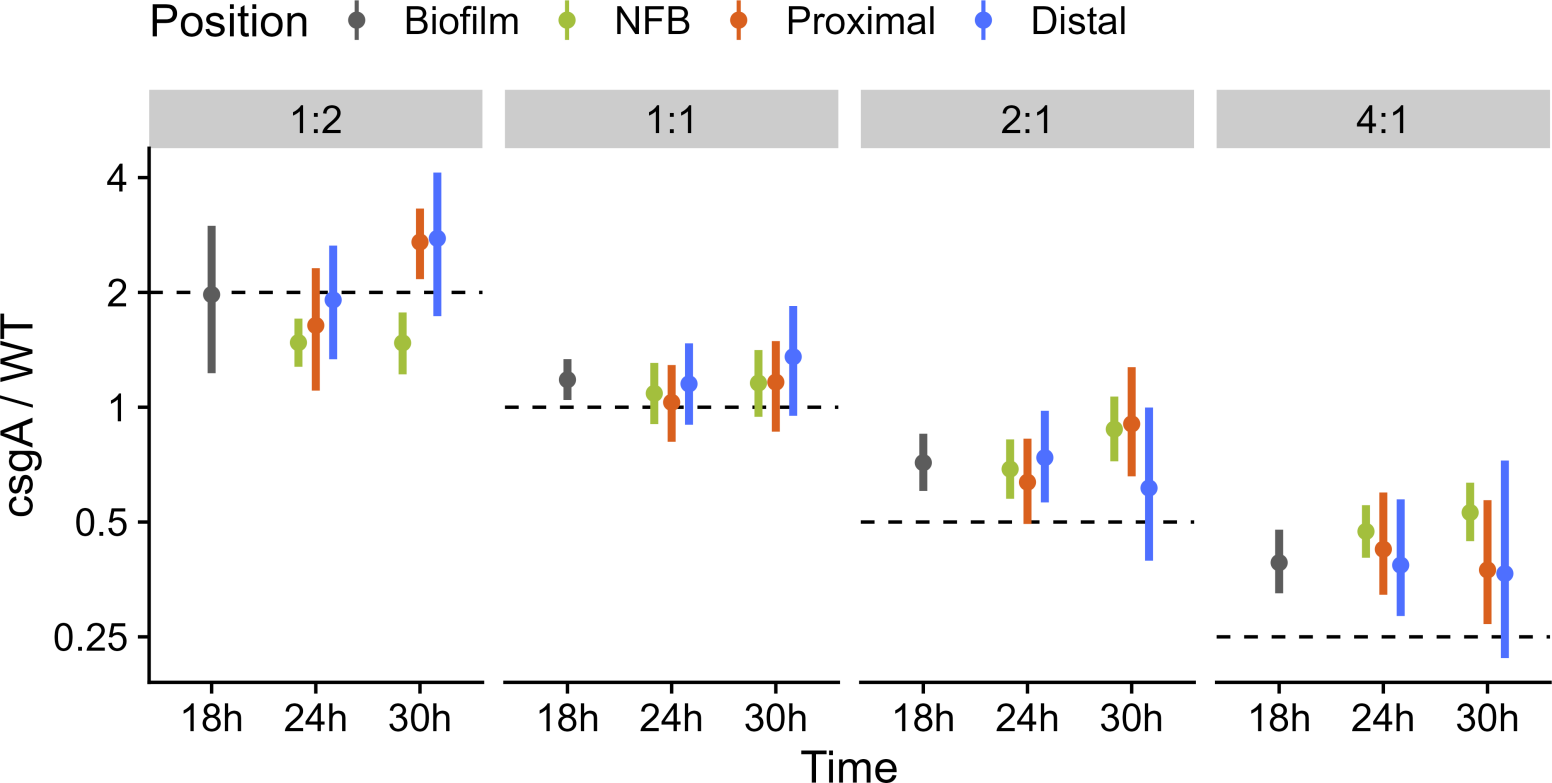
Ratios of *csgA* to wildtype (*csgA*/WT) cells at different times and locations early in development. WT and *csgA* cells were mixed at ratios indicated at the top. The mixtures contained the strains described in the Fig. 1 legend, but each labeled strain was premixed 1:3 (18- and 24-h time points) or 1:1 (30-h time point) with its unlabeled parent. Vanillate (0.5 mM) was added, and the mixtures were starved under submerged culture conditions. *z*-stacks were collected as described in the Fig. 2 legend as follows: distal from any mound (and prior to the formation of most) at 18 h (designated “biofilm”) or at three locations (within a nascent fruiting body [designated “NFB”], “proximal” to the same NFB [one field of view outside], and “distal” from any NFB) at 24 and 30 h. Segmented cells from *z*-stacks of at least five biological replicates were classified as rods, transitioning cells, or spores. Ratios are for totals of all cell classes and were indistinguishable from ratios for only rods, the major cell class. Dot, median. Line, 90% credible interval. Dashed line, initial ratio.

Importantly, for the mixtures initially at ≥2:1, we observed similar ratios of *csgA*/WT indicative of cheating within 24-h NFBs, proximal to the same NFBs (i.e., one field of view outside) and distal from any NFB (Fig. 5). These results are consistent with greater survival of *csgA* than WT throughout the biofilm, rather than greater movement of *csgA* into mounds and/or WT out of mounds. We also found ratios of *csgA*/WT indicative of a similar level of cheating within the 18h biofilm distal from any NFB (before the vast majority of mounds formed) (*P* < 10^−4^), showing that greater survival of *csgA* occurred before mound formation in mixtures initially at ≥2:1. By 30 h PS, the ratios of *csgA*/WT had increased slightly on average within NFBs formed by the 2:1 and 4:1 mixtures (*P* = 0.037 and 0.021, respectively) and proximal to NFBs formed by the 2:1 mixture (*P* = 0.040). However, a positive or negative trend for the *csgA*/WT ratios at the proximal and distal locations could not be determined with enough confidence. Hence, differential survival and/or movement may slightly increase the *csgA*/WT ratios within NFBs by 30 h. We note that the total number of cells in all classes declines from 18 to 30 h at the proximal and distal locations for the mixtures initially at ≥1:1, but the cell number within NFBs remains similar (Fig. S12). However, between 30 and 36 h, the cell number within NFBs declines, especially for WT (Fig. S10), due to lysis of TCs or rods, or rods exiting NFBs, as mentioned above. For the mixture initially at 1:1, the *csgA*/WT ratios were similar at different times and locations (Fig. 5). For the mixture initially at 1:2, at 30 h, the *csgA*/WT ratio was less within NFBs than at the proximal and distal locations (*P* < 10^−2^), consistent with our observation that C-signaling by WT failed to fully rescue participation of *csgA* in mound formation (Fig. 4A). Changing the proportion of labeled to unlabeled cells in the mixtures (i.e., 1:1 vs. 1:3) did not alter mound formation by 24 h (Fig. S3) or the *csgA*/WT ratio at 24 or 30 h (Fig. 4A and 5) for the different initial ratios of WT to *csgA*.

We conclude that cheating occurs before mound formation due to greater survival of *csgA* than WT within 18-h biofilms formed by the mixtures initially at ≥2:1 (Fig. 5). As these mixtures formed mounds by 24 h PS, the overall levels of cheating remained similar within the early NFBs and outside them, but the *csgA* proportion rose near the radial center of NFBs (Fig. 3B), suggesting greater persistence of *csgA* rods there. By 30 h, cheating had increased slightly on average within NFBs (Fig. 5), but cell numbers had declined considerably outside NFBs (Fig. S12), likely contributing to greater uncertainty in the cheating levels. Nevertheless, the high proportion of *csgA* rods persisted near the radial center of NFBs, and later a high proportion of *csgA* spores formed there (Fig. 3B). Although NFBs formed by the mixtures initially at 1:1 did not exhibit cheating by 30 h, a high proportion of *csgA* spores had formed near the radial center by 36 h, accounting for the cheating we observed late in development (Fig. 3B and 4A).

## DISCUSSION

We discovered that mixtures of WT and *csgA* mutant cells at ratios ranging from 1:4 to 4:1 exhibit dramatically different developmental phenotypes in submerged culture. At 1:4, development failed completely. At 1:2, a few mounds formed, but neither WT nor *csgA* made many spores, indicating that C-signaling by the WT minority was insufficient to support normal development of the population. In contrast, when WT comprised half or more of the population, mounds formed normally, and cheating occurred. Interestingly, at 1:1, C-signaling by WT efficiently rescued *csgA* participation in mound building, but cheating did not occur until later, during the sporulation stage of development. Strikingly, at 2:1 or 4:1, *csgA* cheated on C-signaling by the WT majority both before and after mound formation. Greater survival of *csgA* than WT accounted for cheating prior to mound building. Cheating during the sporulation stage involved *csgA* forming spores at a greater rate, while WT disappeared at a greater rate. These new insights into cheating behavior pose important questions for future research. Since cheating by *csgA* required equal or excess WT in the initial mixture, we conclude that short-range C-signaling severely restricts cheating behavior, which likely favored its evolution rather than long-range diffusible signaling. We propose that cheating restrictions imposed by short-range signaling may have likewise proved advantageous during the evolution of bacterial biofilm and multicellular animal development.

### Short-range C-signaling restricts cheating behavior

Excess *csgA* mutant cells in mixtures with WT interfered with development. The mixtures initially at 1:2 and 1:4 showed a progressive and dramatic decrease in mound formation compared with the mixtures at ≥1:1 (Fig. S3), suggesting that *csgA* rods require frequent C-signaling from WT rods to participate in mound building. The inability of *csgA* rods to produce C-signal presumably impairs two positive feedback loops necessary for mound formation. One loop involves the movement of rods into alignment for enhanced C-signaling (41–43). The other loop involves C-signal-dependent transcription of the *act* operon (51), whose products control C-signal production (52). These positive feedback loops depend on the cellular arrangement to enhance C-signaling during mound building. Long-range diffusible signaling could not likewise communicate the cellular arrangement. Rather, it simply communicates the cell density, which presumably is insufficient to build dome-shaped multicellular mounds that become spore-filled fruiting bodies. In the few mounds that formed at 1:2, very few rods became spores (Fig. 3B; Fig. S10), consistent with previous observations supporting that spore formation requires efficient C-signaling (26–28, 47).

Developmental interference by excess *csgA* mutant cells would limit the invasion of WT populations subject to selection for development. Indeed, a mixture of WT and *csgA* cells at 99:1 exhibited cheating by *csgA* during an initial cycle of co-development and co-growth, but *csgA* persisted as the minority with little change in the population dynamics during four subsequent cycles (53). Albeit short in duration, this experimental evolution study supports the notion that cheating restriction imposed by short-range C-signaling likely favored its evolution. In contrast, long-range diffusible signaling, such as secreted “public goods,” are highly susceptible to cheating (13, 15, 54). The restriction of *csgA* cheating to populations with equal or excess WT is reminiscent of the negatively frequency-dependent fitness of two developmental cheaters that evolved from WT clones passaged in liquid culture (14, 55, 56). Further exploration of the frequency-dependent fitness of evolved and defined (e.g., an a*sgB* mutant defective in diffusible A-signaling) (56) cheaters co-developed with WT may shed light on the evolutionary implications of different cheating mechanisms. We speculate that a population-level restriction on cheating behavior strongly favored short-range C-signaling evolutionarily rather than long-range diffusible signaling.

Interestingly, WT can evolve further restrictions on cheating behavior by a *csgA* mutant. WT rapidly evolved cheater suppression and selfish policing during 20 cycles of co-development with *csgA* at 1:1 (34). Even in the absence of *csgA*, WT clones that had evolved as motile colonies on nutrient agar and differed from the ancestral WT by no more than 20 mutations frequently also evolved resistance to developmental cheating by *csgA* (57). The mechanisms of enhanced cheater resistance remain to be elucidated.

Based on our results, we propose that restrictions on cheating behavior were driving forces in the evolution of multicellularity and explain the prevalence of short-range signaling in bacterial biofilm and animal development. Cheating during animal development can cause tumor formation and lead to metastatic cancer (13, 58). Defects in short-range signaling mechanisms, such as those involving growth factors (59, 60), Hedgehog (61), Wnt (62), and Notch (63), are often associated with carcinogenesis, suggesting that restrictions on cheating behavior by these mechanisms may have selected their use and explain their prevalence in animal development. Short-range signaling is likewise prevalent in bacterial biofilm development (6–8). Numerous examples of kin selection mediated by cell–cell contact-dependent mechanisms restrict cheating in biofilms (6, 8, 9), including in *M. xanthus* (64–67). The extracellular matrix and environmental forces also restrict cheating by spatially structuring biofilms, thus limiting cell dispersal and diffusion of public goods and signals with potential longer range (8, 9).

### Cheating before mound formation involves greater survival of the *csgA* mutant in mixtures with excess wild-type cells

Excess WT cells in mixtures initially at ≥2:1 allowed cheating by *csgA* before mound formation owing to greater survival of *csgA* than WT by 18 h PS (Fig. 5). Greater survival of unmixed developing *csgA* rods compared with WT has been reported previously (46, 48–50), suggesting a partial defect in developmental lysis of *csgA*. The mechanism of developmental lysis is unknown (49, 68). Interestingly, we did not observe greater survival of *csgA* than WT at 18 h in the mixture initially at 1:2, and *csgA* barely outnumbered WT in the mixture initially at 1:1 (Fig. 5). Taken together, these observations suggest that an initial excess of WT promotes greater *csgA* survival in mixtures, but equal or less WT inhibits *csgA* survival (Fig. 5). Perhaps, components of living and/or lysed WT cells exert different concentration-dependent effects on *csgA*, whose stringent response to starvation differs from that of WT (69). Further elucidating differences between *csgA* and WT in the stringent response, lysis, and signaling will be important for deeper understanding of *csgA* cheating behavior in mixtures prior to mound formation and likely later in development as well.

Cheating during mound formation generates spatial differentiation within NFBs. We observed a high proportion of *csgA* rods at 24 and 30 h PS near the radial center of NFBs formed by mixtures initially at ≥2:1, and a high proportion of *csgA* spores there later for mixtures initially at ≥1:1 (Fig. 3B). Presumably, this pattern forms *via* differential survival and/or movement. We favor differential survival as the explanation for the high proportion of *csgA* spores near the radial center of 36-h NFBs formed by mixtures initially at 1:1, since we did not observe a high proportion of *csgA* rods there earlier. However, our results do not exclude the possibility of differential movement of *csgA* and WT rods between 30 and 36 h. Neither do our results exclude differential movement of *csgA* and WT rods as the explanation for the high proportion of *csgA* near the radial center of 24-h NFBs formed by mixtures initially at ≥2:1. Our confocal microscopy method is currently incompatible with tracking of individual cells within NFBs (see below), but our comparison of the overall level of cheating within 24-h NFBs to proximal and distal locations outside revealed little or no difference (Fig. 5). Moreover, the cheating levels were similar to those in the 18-h biofilms, so a similar ability of *csgA* and WT rods to move into mounds and persistence there could account for the similar level of cheating within and outside of NFBs. In agreement, the accumulation rates of *csgA* and WT within mounds were indistinguishable in cell tracking experiments (44, 70). The detailed motility behavior of *csgA* and WT rods differed, but spatial differentiation within the mounds was not reported. The cell tracking method used a small fraction of labeled trackable cells (≤0.04%) and frequent (every 30 s for ~5 h), brief (600 ms) imaging. In contrast, our method is currently incompatible with tracking since we have used 17%–50% labeled cells and infrequent, lengthy imaging (4 *z*-stacks, ~2.5 min each, 3–6 h apart) (47). It may be possible to use a cell tracking method (44, 70), and the insights about cheating behavior revealed by our study to determine the contributions of differential survival and movement to spatial differentiation within NFBs formed by mixtures of *csgA* and WT.

### Cheating after mound formation involves more efficient sporulation of the *csgA* mutant in mixtures with equal or excess wild-type cells

When WT comprised half or more of the population, cheating occurred during the sporulation stage of development (Fig. 3 and 4A) and involved *csgA* forming spores at a greater rate, while WT disappeared at a greater rate (Fig. 4B; Fig. S10). Why does *csgA* form spores more efficiently than WT? There are many possible reasons. The altered stringent response of *csgA* (69) may enhance its protein synthesis capacity (relative to WT), improving its sporulation efficiency. The *csgA* mutant lacks cardiolipin phospholipase activity (40) and fails to synthesize lipid bodies or undergo cell shortening (71), so its lipid and membrane metabolism differ from WT, as does its developmental gene expression (35, 46, 72). C-signaling from WT may trigger efficient sporulation of *csgA* without completely restoring normal metabolism and/or gene expression, perhaps akin to chemically induced sporulation of *M. xanthus* (73, 74). This deserves further investigation. Importantly, our data show that *csgA* cheating occurs early in the sporulation stage, primarily between 30 and 36 h (Fig. 3 and 4; Fig. S10), much earlier than tested in previous studies (33, 34, 53, 56, 57). Our results imply that *csgA* rods are poised to efficiently transition to spores upon sensing WT C-signaling in NFBs.

Why does WT disappear from NFBs at a greater rate than *csgA*? Under our conditions of submerged culture development, unmixed WT cells appear to lyse more rapidly than unmixed *csgA* cells at 18–48 h PS (46), so it is possible that differential lysis contributes to the greater disappearance of WT than *csgA* during the spore-forming stage of development (Fig. 3 and 4; Fig. S10). We cannot confidently rule out the possibility that WT rods preferentially exit NFBs after 30 h. There was no evidence of preferential WT exiting to proximal or distal locations at 30 h, but the uncertainty was great (Fig. 5) likely due to low cell numbers, which decline further by 36 h near the edge of NFBs (Fig. 1, 2A and 3A) and in samples of the entire biofilm (46).

In summary, we conclude that short-range C-signaling restricts cheating in mixtures with a WT minority, cheating occurs only during the spore-forming stage in mixtures with equal WT and *csgA* cells, and cheating occurs during both the mound- and spore-forming stages in mixtures with a WT majority.

## MATERIALS AND METHODS

### Bacterial strains and plasmids

Table S1 lists the strains, plasmids, and primers used in this study. The construction of *M. xanthus* strain YH11 is described in the Supplemental Material.

### Growth and development

*M. xanthus* was grown and submerged culture development was performed as described in the Supplemental Material.

### Microscopy

Images were acquired with laser scanning confocal microscopes and fluorescence from tdTomato and mNeonGreen was examined as described in the Supplemental Material.

### Image analyses

Cell segmentation, cellular morphology classification, and estimation of the proportions of cell classes across time points and along the radii of NFBs were performed as described previously (47) with the following modifications to improve the sensitivity of cell detection. Otsu’s method as implemented in MATLAB was used on the center quarter of each image to determine the optimal threshold value to binarize the entire images after calculating the Hessian matrix, instead of using the 99th percentile. Because the new threshold slightly increased the apparent cell volume after segmentation, the Gaussian mixture model used to classify cells into morphological types was refitted following the original protocol. To estimate cell density in the stack of images, the number of labeled cells per 1,000 µm^3^ was doubled to account for unlabeled cells in the mixture, or quadrupled for the 18- and 24-h time points in Fig. S12. Plots were generated in the R statistical environment using the ggplot (75) and tidybayes (76) packages.

## Data Availability

Confocal image stacks are available in Dryad (DOI: 10.5061/dryad.tmpg4f51d). The data and code for the analyses presented in the figures are available in Gitlab (https://gitlab.msu.edu/kroos/segment_myxo_confocal_csgA).

## SUPPLEMENTAL MATERIAL

Supplemental material mbio.02440-24-s0001.pdf Supplemental methods, table, and figures.

ASM does not own the copyrights to Supplemental Material that may be linked to, or accessed through, an article. The authors have granted ASM a non-exclusive, world-wide license to publish the Supplemental Material files. Please contact the corresponding author directly for reuse.
