# Supplementary material for: Short-range C-signaling restricts cheating behavior during Myxococcus xanthus development
Source: mBio. 2024 Oct 18;15(11):e02440-24. doi: 10.1128/mbio.02440-24 (PMC11559036; doi:10.1128/mbio.02440-24)
Supplement: Supplemental material — Supplemental methods, table, and figures. [file mbio.02440-24-s0001.pdf]

SUPPLEMENTAL MATERIAL

Short-Range C-Signaling Restricts Cheating Behavior during *Myxococcus xanthus* Development

Y Hoang[1, 2], Joshua Franklin[2], Yann S. Dufour[2 #], and Lee Kroos[1, 2 #]

[1]Department of Biochemistry and Molecular Biology, Michigan State University, East Lansing, Michigan, USA

[2]Department of Microbiology and Molecular Genetics, Michigan State University, East Lansing, Michigan, USA

MATERIALS AND METHODS

Construction of *M. xanthus* strain YH11. To construct *M. xanthus* YH11, pYH8 was transformed into *M. xanthus* MRR33 using electroporation (1), with selection for tetracycline resistance (15 μg/mL) on Casitone-Tris (CTT) (1% Casitone, 10 mM Tris-HCl [pH 8.0], 1 mM KH_2PO_4-K_2HPO_4, 8 mM $MgSO_4$, [final pH 7.6]) agar (1.5%). Integration of pYH8 was verified by colony PCR with primers pMR3691 MCS G-F and pMR3691 MCS G-R.

Growth and development. *M. xanthus* was grown and submerged culture development was performed as described previously (2). Briefly, cells were grown at 32°C in CTTYE liquid medium (CTT with 0.2% yeast extract) with shaking at 350 rpm. Development was performed in 8-well μ-slides (Ibidi #80826) with starvation buffer MC7 (10 mM morpholinepropanesulfonic acid [MOPS, pH 7.0], 1 mM $CaCl_2$). Cells from log-phase CTTYE cultures were collected by centrifugation and resuspended to 1,000 Klett units in MC7 containing 0.5 mM vanillate. The cell suspensions were mixed as described in the text and the figure legends. Each cell suspension (30 μL) was then added to 201 μL of MC7 containing 0.5 mM vanillate, and 200 μL was pipeted into each μ-slide well. The μ-slide was wrapped in clear plastic and incubated at 32°C in a humid chamber.

Microscopy. Images of NFBs were acquired with a Nikon A1 Laser Scanning Confocal Microscope and fluorescence from tdTomato and mNeonGreen was examined as described previously (2). Briefly, the microscope was configured on a Nikon Ti inverted platform with an XY automated stage. Brightfield and fluorescence images were acquired with 10X and 100X objectives, respectively. Fluorescence from tdTomato was examined using a 560-nm laser for excitation and a 595/50 band pass emission filter. Fluorescence from mNeonGreen was examined using a 488 nm laser for excitation and a 525/50 band pass emission filter. Images near the bottom of NFBs were the first optical section above the bottom of the well, in which cells could be clearly visualized, so ~0.25 to 0.5 μm above the bottom of the well.

To compare the observed to expected ratios of initial cell mixtures, cells were grown as described above, except in the presence of vanillate (0.5 mM). Cells were collected by centrifugation, resuspended, and mixed as described above. Unmixed cells served as controls to determine settings that minimized autofluorescence yet ensured that > 90% of labeled cells were counted. Each cell suspension (10 µL) was added to 40 µL of MC7 containing 0.5 mM vanillate, then 10 µL was pipeted onto a microscope slide and covered with a 24 x 50 mm coverslip. Images of cells were acquired with a Nikon C2+ Laser Scanning Confocal Microscope configured on a Nikon Eclipse Ni upright platform. DIC transmitted light and fluorescence images were acquired with a 60x Plan Apo oil objective (NA 1.40). Fluorescence from tdTomato was examined using a 561-nm laser for excitation and a 600/50 band pass emission filter. Fluorescence from mNeonGreen was examined using a 488 nm laser for excitation and a 525/50 band pass emission filter. Images were collected for three fields of view for each biological replicate and cells exhibiting green or red fluorescence were counted manually.

Table S1. Bacterial strains, plasmids, and primers used in this study

**Strain or plasmid**	**Description**	**Source or reference**
**Strain**		
E. coli		
DH5α	λ^- ϕ80d*lacZΔM15* Δ(*lacZYA-argF*)*U169 recA1 endA1 hsdR17*($r_K^-m_K^-$) *supE44 thi-1 gyrA relA1*	(3)
M. xanthus		
DK1622	Laboratory wild-type strain	(4)
MRR33	*csgA*::pRR028	(5, 6)
YH7	DK1622 with MXAN_0018-MXAN_0019::pYH7	(2)
YH11	MRR33 with MXAN_0018-MXAN_0019::pYH8	This study
**Plasmid**		
pYH7	MXAN_0018-MXAN_0019-$P_{R3\text{-}4}$::*vanR*-P_{van}::*mNeonGreen*, Tet^r	(2)
pYH8	MXAN_0018-MXAN_0019-$P_{R3\text{-}4}$::*vanR*-P_{van}::*tdTomato*, Tet^r	(2)
**Primer**		
pMR3691 MCS G-F	CACGATGCGAGGAAACGCA	This study
pMR3691 MCS G-R	CACCGGTACGCGTAACGTTC	This study

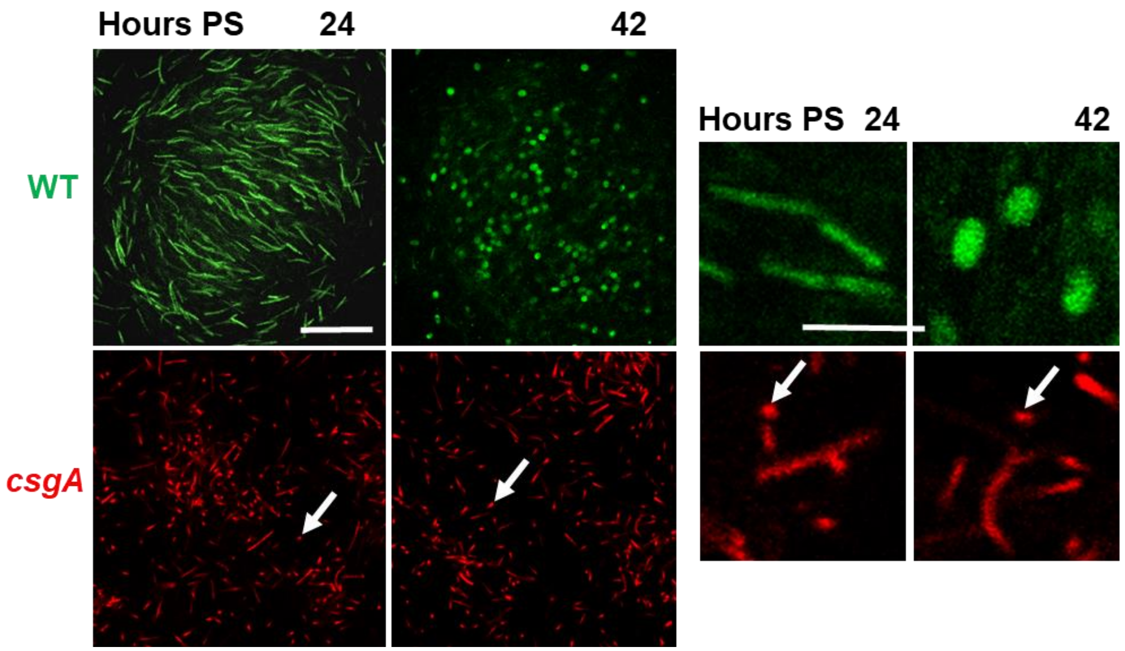

FIG S1 Development of wild type (WT) and *csgA* mutant cells separately. The top row of panels shows labeled WT strain YH7 (green fluorescence) mixed with unlabeled WT strain DK1622 at a ratio of 1:5. The bottom row of panels shows labeled *csgA* strain YH11 (red fluorescence) mixed with unlabeled *csgA* strain MRR33 at a ratio of 1:5. Vanillate (0.5 mM) was added and the mixtures were starved under submerged culture conditions. Confocal images of the same field of view were acquired near the bottom (i.e., the first optical section above the bottom of the well in which cells could be clearly visualized, so ~0.25 to 0.5 μm above the bottom of the well) of the biofilm or a mound at the indicated times poststarvation (PS). Arrows indicate small dots of red fluorescence, due to cross-sectional views of *csgA* rods oriented vertically, based on analysis of *z*-stacks of optical sections. Panels on the left and right are at lower (bar, 20 μm) and higher (bar, 5 μm) magnification, respectively.

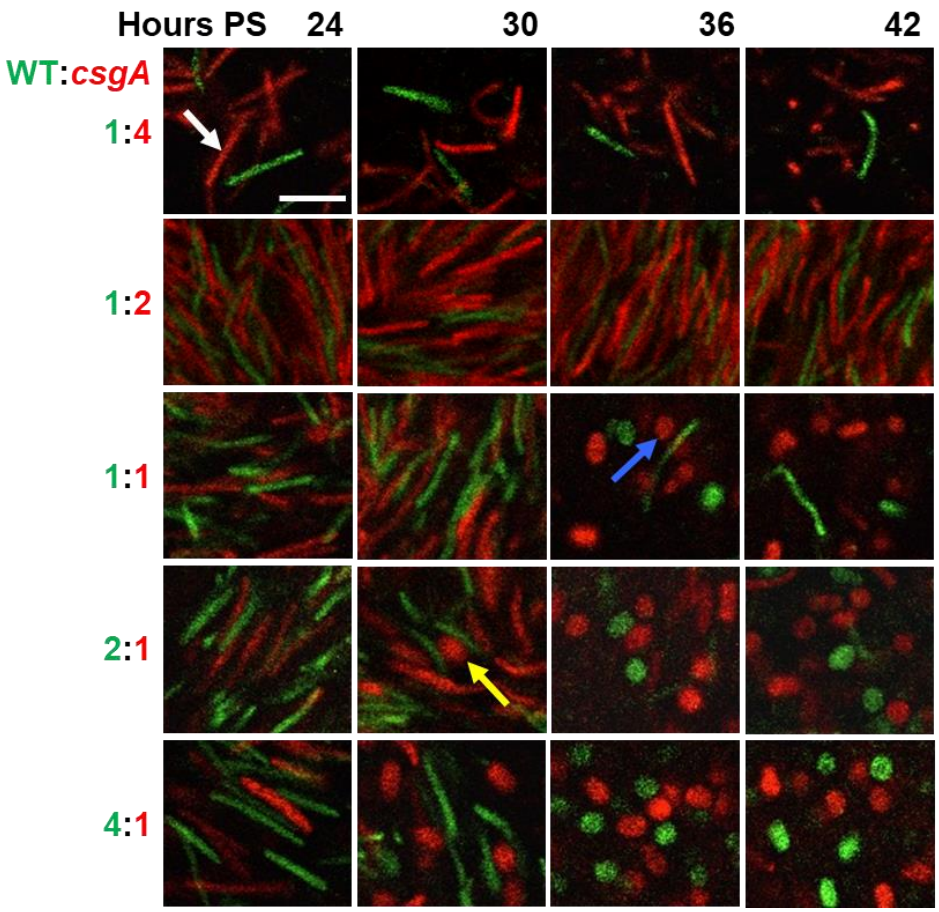

FIG S2 Images of representative cells during co-development at different ratios. Higher magnification images from Fig. 1 show *csgA* mutant cells with a particular shape. PS, poststarvation. WT, wild type. White arrow, rod. Yellow arrow, transitioning cell. Blue arrow, spore. Bar, 5 μm.

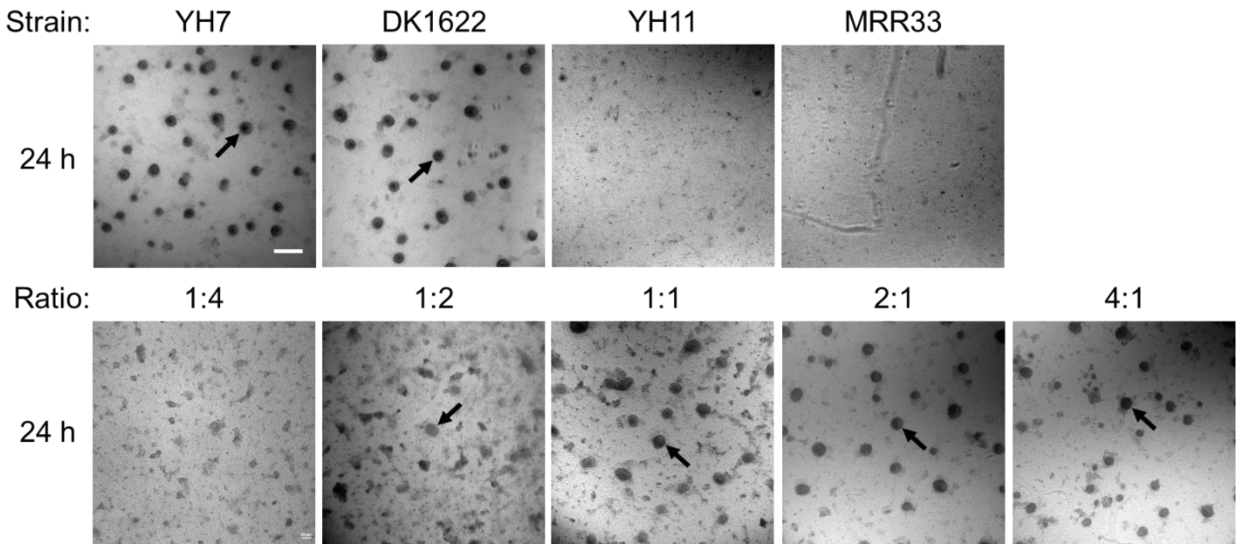

FIG S3 Development of individual strains and mixtures. The strains and mixtures are described in the Fig. 1 legend. Vanillate (0.5 mM) was added and the cells were starved under submerged culture conditions. Bright-field images were acquired at 24 h poststarvation. Arrows, mounds. Bar, 200 μm. The results were similar if the proportion of labeled to unlabeled cells in the mixtures was 1:3 as described in the Fig. 5 legend, rather than 1:1.

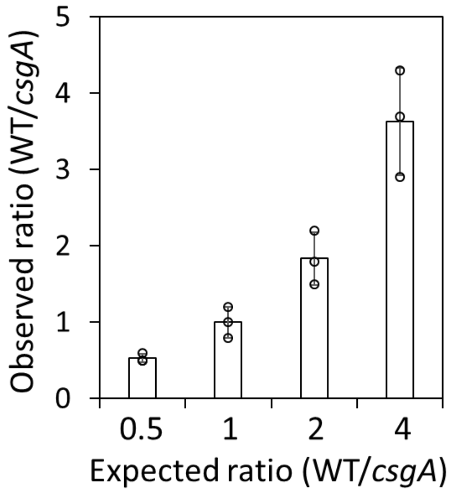

FIG S4 Comparison of observed to expected ratios of initial cell mixtures. The strains and mixtures are described in the Fig. 1 legend, but in this experiment the strains were grown in the presence of vanillate (0.5 mM) and labeled cells in each mixture were counted for green or red fluorescence. The observed ratio is green wild type (WT) cells/red *csgA* cells. Three biological replicates were performed and > 400 cells total were counted for each expected ratio. Bar, average. Circles, replicates. Error bar, one standard deviation.

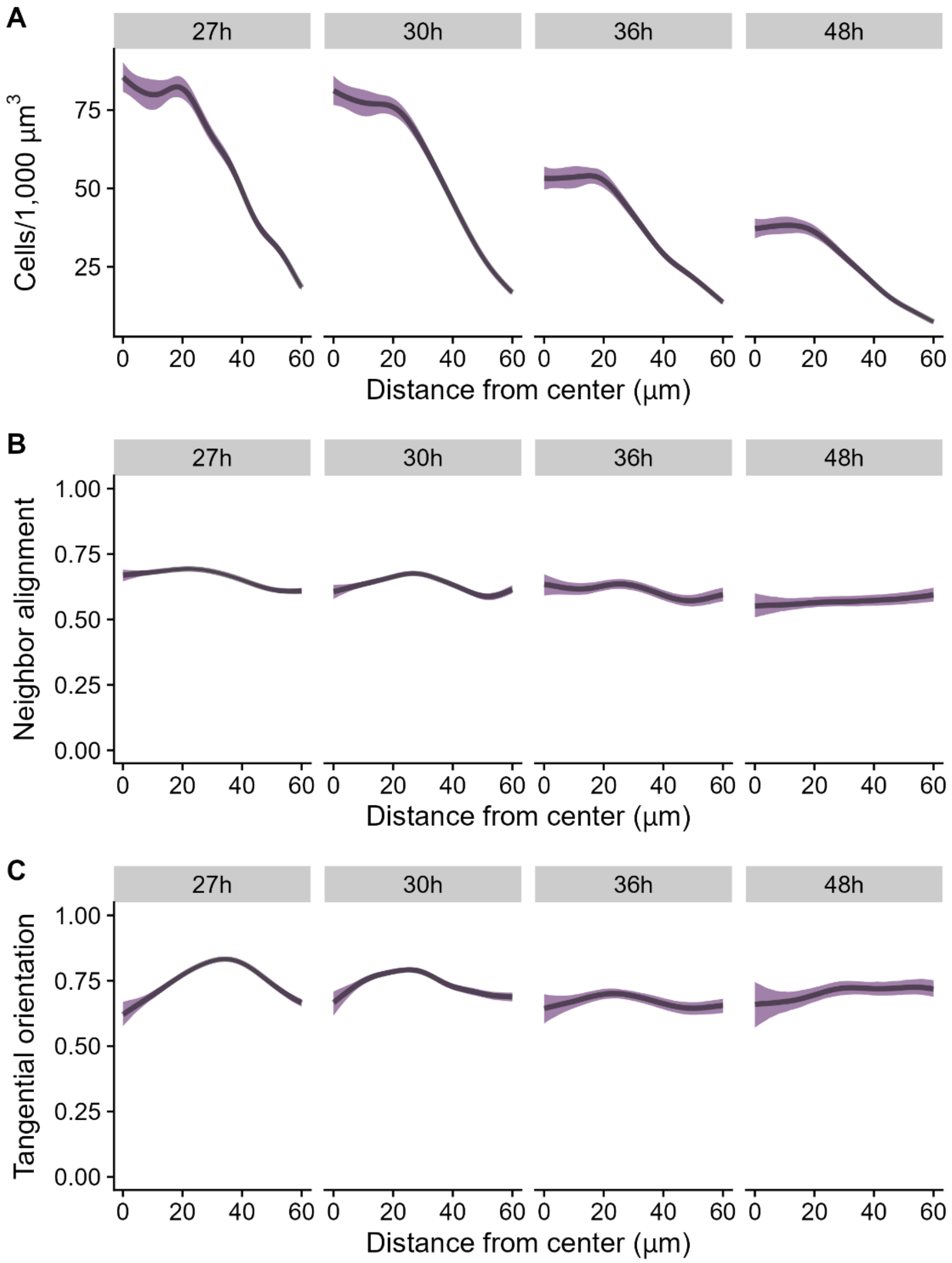

FIG S5 Radial patterns of cell density, neighbor alignment, and tangential orientation for wild type. In experiments described previously (2), labeled WT strains (YH7, YH8, YH14, or YH15) were mixed with unlabeled WT strain DK1622 at a ratio of 1:5. Vanillate (0.5 mM) was added and the mixtures were starved under submerged culture conditions. Confocal images of the same field of view were acquired at the indicated times poststarvation near the bottom of the well as described in the Fig. 1 legend. Segmented cells from *z*-stacks of nascent fruiting bodies (NFBs) from five biological replicates for each mixture (i.e., 20 NFBs total) were classified as rods, transitioning cells, or spores using a modified computational analysis with improved cell segmentation and classification. Cell density (A), neighbor alignment of rods (B), and tangential orientation of rods (C) were determined as described in the Fig. 2 legend. Line, median. Shaded region, 90% credible interval.

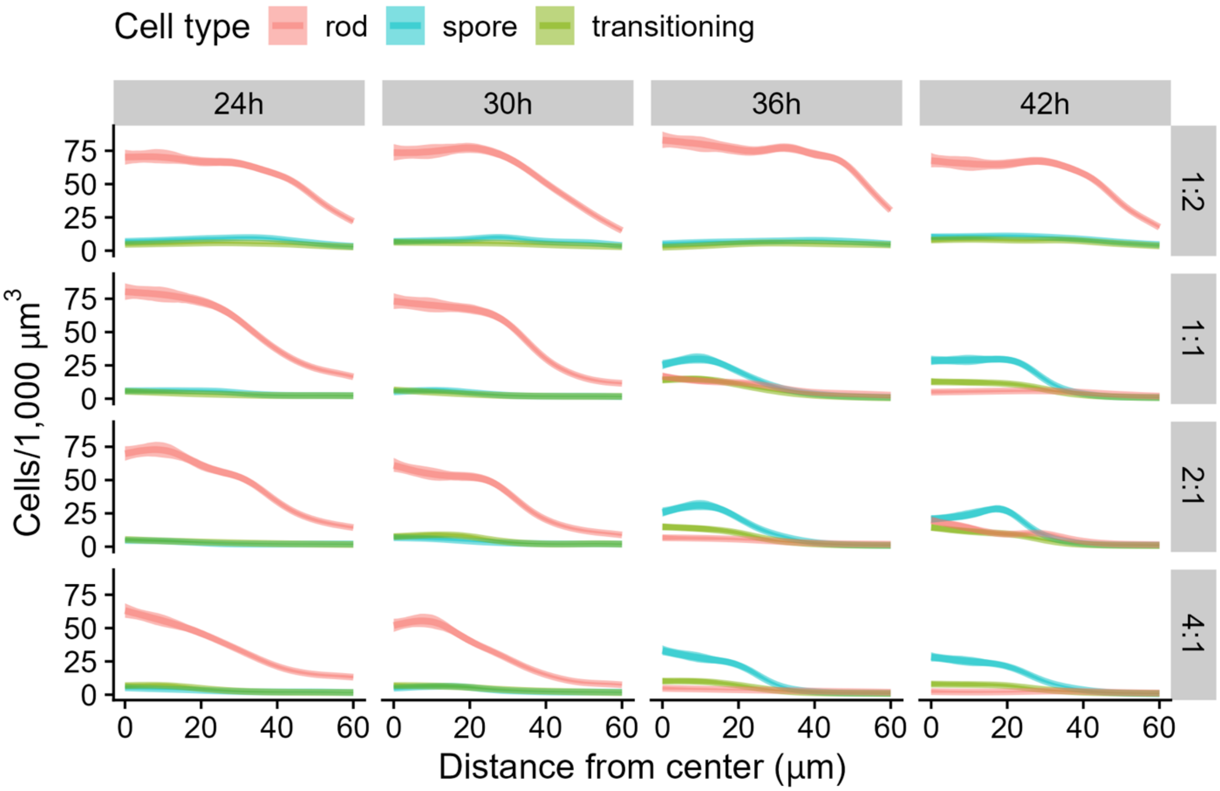

FIG S6 Radial patterns of cell density of different cell classes in co-developed mixtures. In the experiment described in the Fig. 1 legend, segmented cells from all *z*-stacks were classified as rods, spores, or transitioning cells (transitioning). The combined results for both wild type (WT) green- and *csgA* red-labeled cells are shown. Cell density of each cell class from the center (0 µm) to the edge (60 µm) of nascent fruiting bodies at different times poststarvation. Line, median. Shaded region, 90% credible interval. WT and *csgA* cells were mixed initially at the ratios indicated on the right.

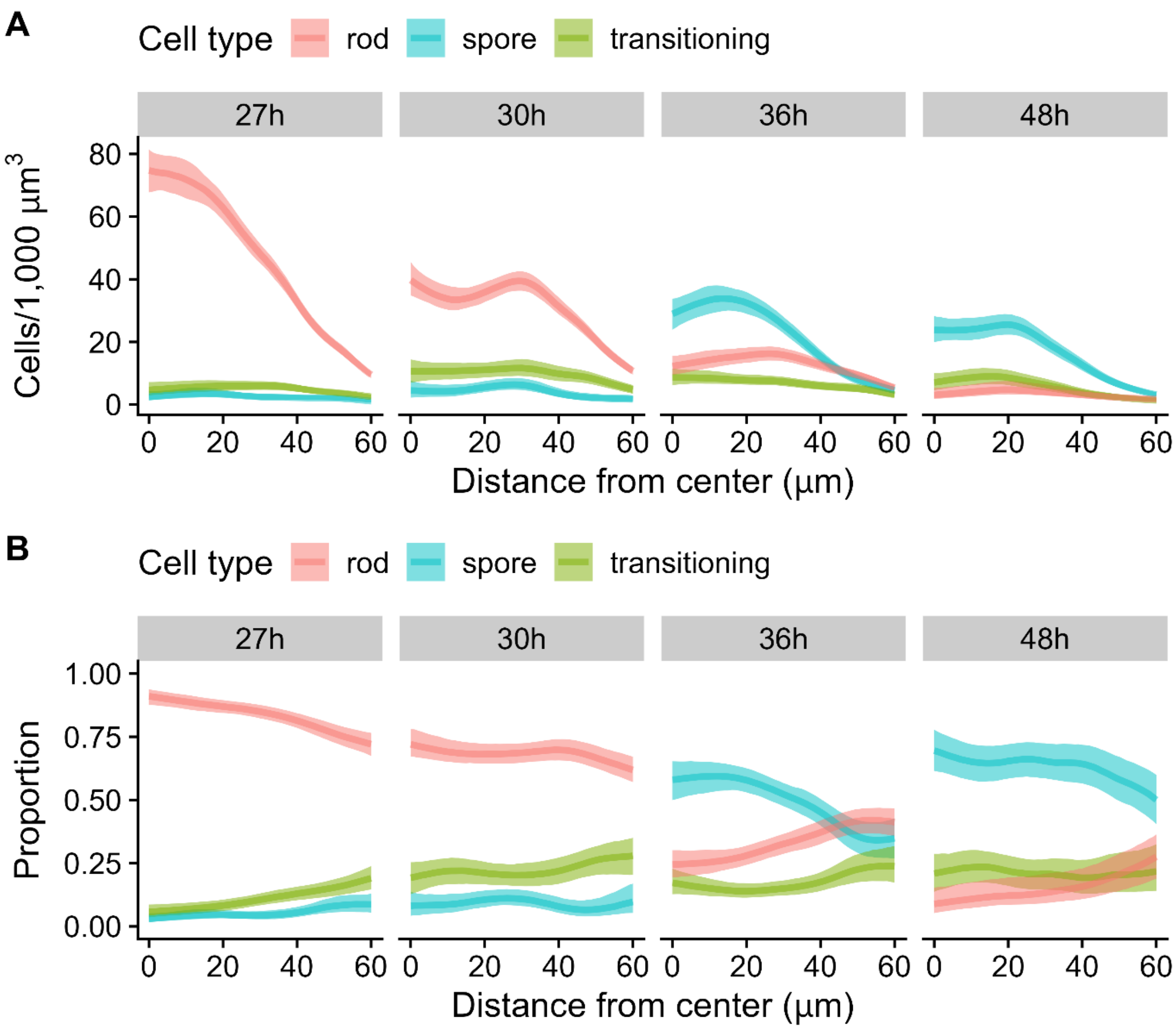

FIG S7 Radial patterns of wild type rods, transitioning cells (transitioning), and spores during development. Data from experiments described previously (2) and in the Fig. S5 legend were analyzed to determine cell density (A) and proportion (B) as described in the Fig. 3 legend. Line, median. Shaded region, 90% credible interval.

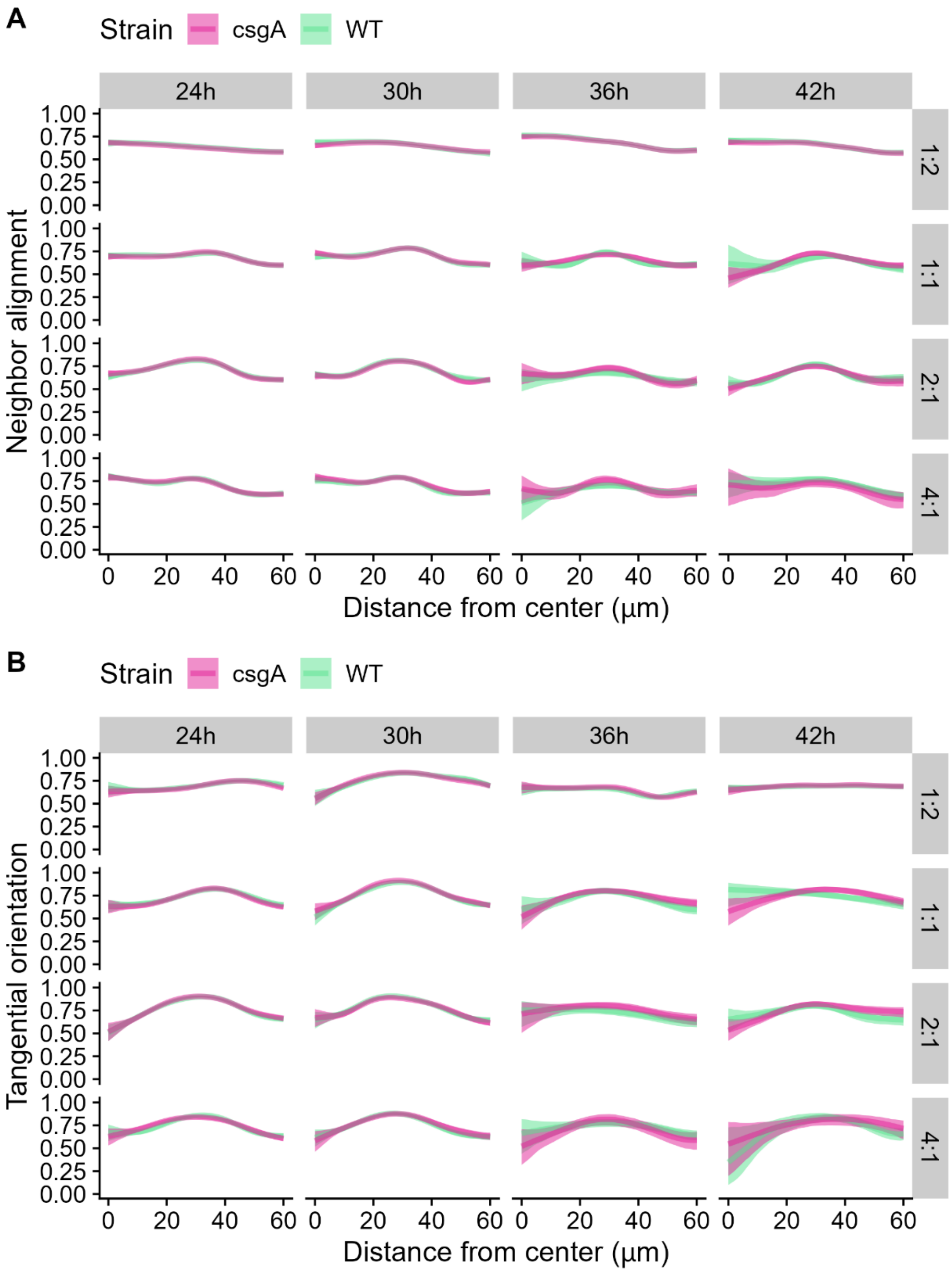

FIG S8 Radial patterns of neighbor alignment and tangential orientation of wild type (WT) and *csgA* mutant rods in co-developed mixtures. In the experiment described in the Fig. 1 legend, segmented cells from all *z*-stacks were classified as rods, transitioning cells, or spores. (A) Neighbor alignment of rods from the center (0 µm) to the edge (60 µm) of nascent fruiting bodies (NFBs) at different times poststarvation. Alignment is defined in the Fig. 2 legend. (B) Tangential orientation of rods radially in NFBs over time. Orientation is defined in the Fig. 2 legend. Line, median. Shaded region, 90% credible interval. WT and *csgA* cells were mixed initially at the ratios indicated on the right.

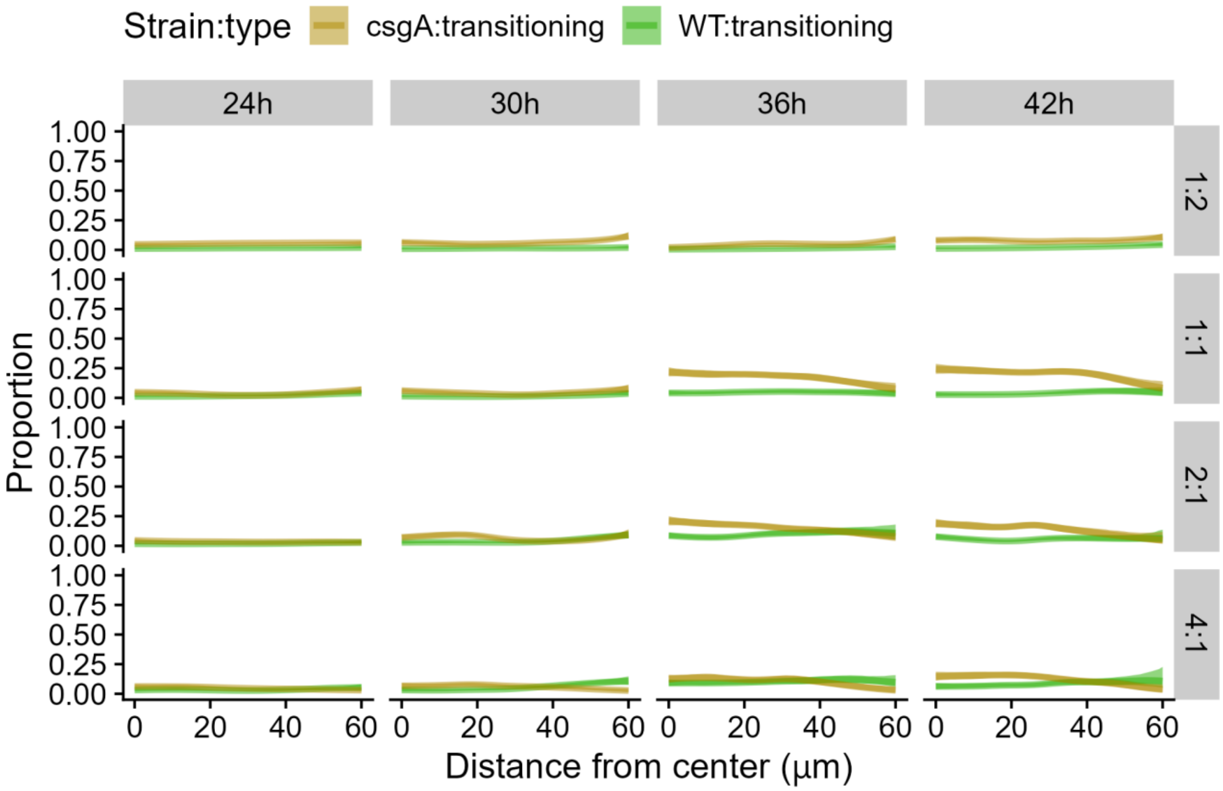

FIG S9 Radial patterns of wild type (WT) and *csgA* transitioning cells (transitioning) in co-developed mixtures. In the experiment described in the Fig. 1 legend, segmented cells from all *z*-stacks were classified as rods, transitioning cells, or spores. Proportion of WT and *csgA* transitioning cells (relative to the combined total number of cells in all classes for both strains) from the center (0 µm) to the edge (60 µm) of NFBs at different times poststarvation. Line, median. Shaded region, 90% credible interval. WT and *csgA* cells were mixed initially at the ratios indicated on the right.

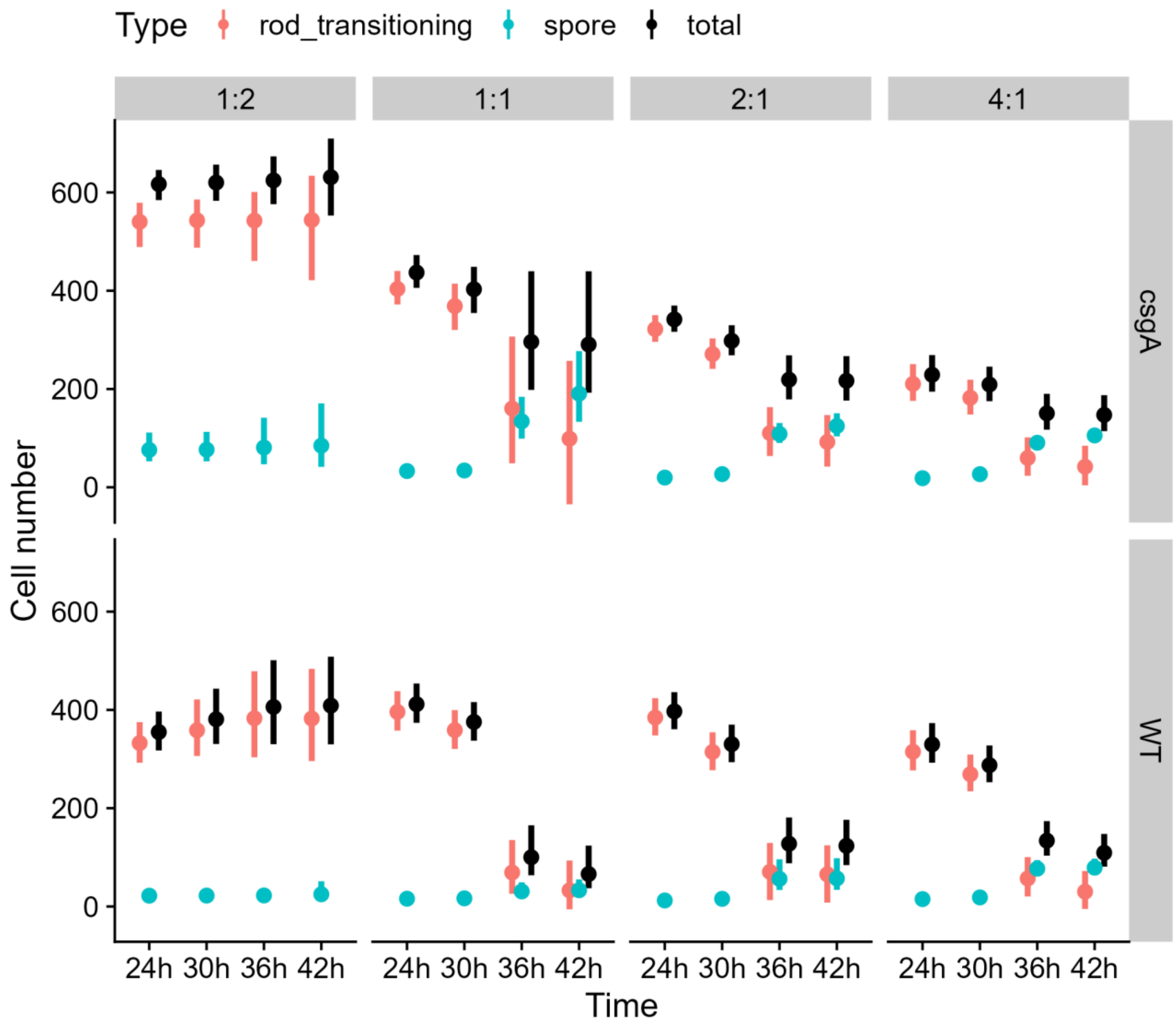

FIG S10 Numbers of wild type (WT) and *csgA* cells in co-developed mixtures over time. In the experiment described in the Fig. 1 legend, segmented cells from all *z*-stacks were classified as rods, transitioning cells, or spores. Cells within 60 μm of the radial center of nascent fruiting bodies were counted. The numbers of rods plus transitioning cells (rod_transitioning), spores, and total cells at indicated times poststarvation are shown. Dot, median. Line, 90% credible interval.

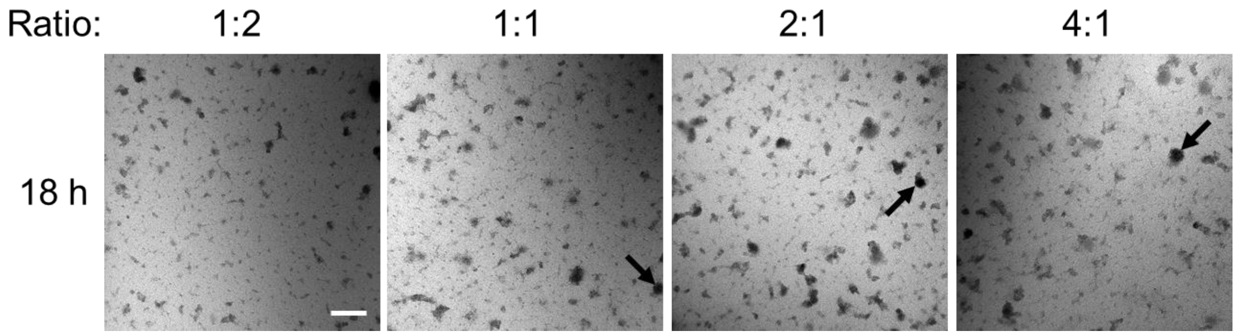

FIG S11 Co-development of mixtures. The strains and mixtures are described in the Fig. 1 and 5 legends, respectively. Vanillate (0.5 mM) was added and the cells were starved under submerged culture conditions. Bright-field images were acquired at 18 h poststarvation. Arrows, mounds. Bar, 200 μm.

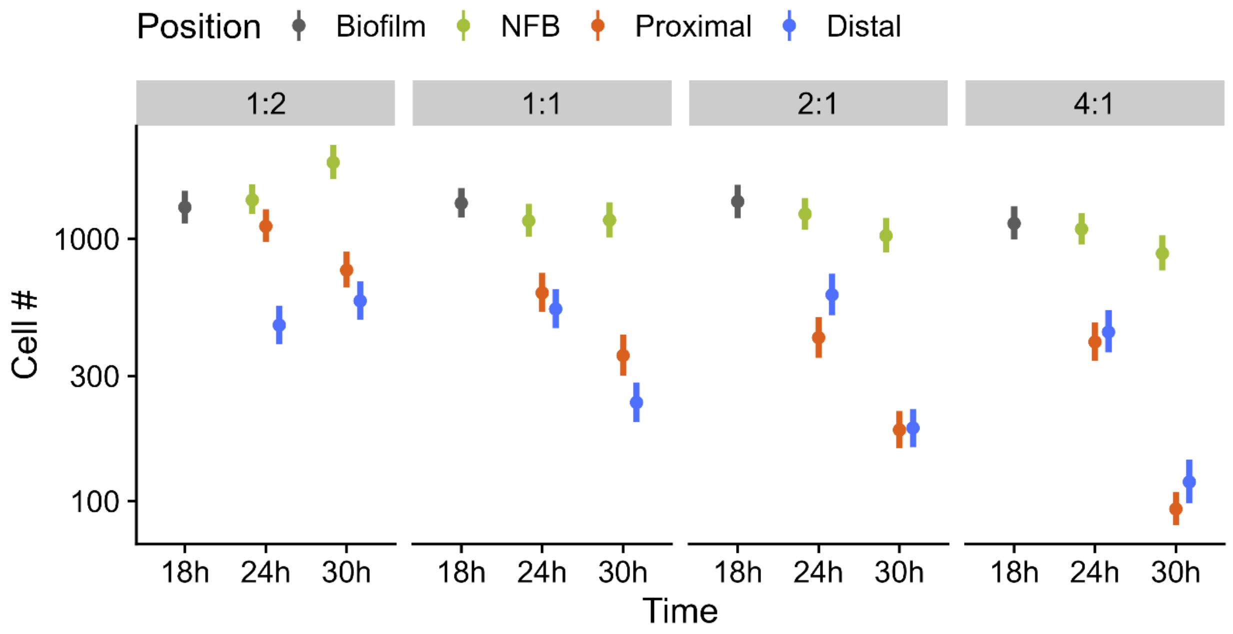

FIG S12 Cell numbers at different times and locations early in development. In the experiment described in the Fig. 5 legend, segmented cells from *z*-stacks of at least five biological replicates were classified as rods, transitioning cells, or spores. The graph shows the total number of cells in all classes. Dot, median. Line, 90% credible interval.

REFERENCES

1. **Kashefi K, Hartzell P.** 1995. Genetic supression and phenotypic masking of a *Myxococcus xanthux frzF*⁻ defect. Molec. Microbiol. **15:**483-494.
2. **Hoang Y, Franklin JL, Dufour YS, Kroos L.** 2021. Cell density, alignment, and orientation correlate with C-signal-dependent gene expression during *Myxococcus xanthus* development. Proc. Natl. Acad. Sci. USA **118:**e2111706118.
3. **Hanahan D.** 1983. Studies on transformation of *Escherichia coli* with plasmids. J. Mol. Biol. **166:**557-580.
4. **Kaiser D.** 1979. Social gliding is correlated with the presence of pili in *Myxococcus xanthus*. Proc. Natl. Acad. Sci. USA **76:**5952-5956.
5. **Rajagopalan R, Kroos L.** 2017. The *dev* operon regulates the timing of sporulation during *Myxococcus xanthus* development. J. Bacteriol. **199:**e00788-00716.
6. **Saha S, Patra P, Igoshin O, Kroos L.** 2019. Systematic analysis of the *Myxococcus xanthus* developmental gene regulatory network supports posttranslational regulation of FruA by C-signaling. Mol. Microbiol. **111:**1732-1752.
